# Editorial: The role of front-of-pack labeling in making informed and healthy food choices

**DOI:** 10.3389/fnut.2023.1231977

**Published:** 2023-07-18

**Authors:** Daniela Martini, Jordi Salas-Salvadó, Mauro Serafini

**Affiliations:** ^1^Department of Food, Environmental, and Nutritional Sciences (DeFENS), Università Degli Studi di Milano, Milan, Italy; ^2^Unitat de Nutrició Humana, Departament de Bioquímica i Biotecnologia, Universitat Rovira i Virgili, Reus, Spain; ^3^Institut d'Investigació Sanitària Pere Virgili (IISPV), Hospital Universitari Sant Joan de Reus, Reus, Spain; ^4^CIBER Fisiopatología de la Obesidad y Nutrición (CIBERObn), Instituto de Salud Carlos III, Madrid, Spain; ^5^Faculty of Biosciences and Technologies for Agriculture, Food, and Environment, University of Teramo, Teramo, Italy

**Keywords:** front-of-pack labeling, food choices, eating behavior, consumer acceptance, health

Food labeling should help consumers in making informed and possibly healthy food choices. For this reason, the labeling of most prepacked foods sold in Europe reports information that should help consumers to make conscious food choices. This information includes: (i) mandatory particulars listed in the Reg. (EU) n.1169/2011), such as the list of ingredients, the presence of allergens, the date of minimum durability or the “use by” date and the nutrition declarations; (ii) voluntary information, such as nutrition and health claims as defined by the Reg. (CE) n.1924/2006). Moreover, in compliance with Art. 35 of the Reg. (EU) n. 1169/2011, many European countries have developed additional forms of expression and presentation of this information, to be reported in the “front-of-pack” (FOP) with the intention to integrate the nutritional information in the principal field of vision of the food and drink pack.

So far, many FOP schemes have been developed – and in some cases already used - in Europe, including both nutrient-specific labels such as Reference Intake labels and NutrInform battery, and summary indicators like Keyhole and Nutri-Score. Also outside Europe, other FOP schemes have been proposed (e.g., warning label and Health Star Rating System).

More recently, the European strategic program “Farm to Fork” has been released with the intention to reach the consensus for a harmonized FOP label proposal. This has fostered the publication of a large body of literature that aimed to better elucidate the acceptance and understanding to some FOP schemes in various groups of the population, as well as to estimate the impact of FOP on food purchases, food habits and in turn health, and finally to investigate if and how the implementation of a FOP scheme can stimulate food companies to reformulate food products.

Although many publications have explored these aspects in the last years, there is still a strong need for a scientific discussion on this topic to better understand the possible impact of FOP in reducing the burden of obesity and related chronic diseases by helping consumers in making better food choices and in adhering to healthy and sustainable dietary patterns. The aim of this Research Topic was therefore to provide a platform for a scientific debate about FOP labeling.

The manuscript by Khoury et al. prospectively assessed the association between the modified version of the Food Standard Agency Nutrient Profiling System Dietary Index (FSAm-NPS DI) that underpin the Nutri-Score FOP and some risk factors for cardiovascular diseases (CVD). Authors observed that participants with a higher FSAm-NPS DI (i.e., corresponding to a lower quality of their diet), showed a significant increase in the levels of CVD markers such as plasma glucose, triglycerides, diastolic blood pressure, and waist circumference.

Bullón-Vela et al. evaluated the association between the nutrient profile underlying the Chilean warning label score and all-cause mortality, observing that a higher score in the warning label values (i.e., lower nutritional quality), was associated with an increased risk of all-cause mortality and cancer mortality during the 12 years of follow-up.

Martini et al. used three different FOP proposed in the European Union (i.e., Nutri-Score, Keyhole and NutrInform Battery) to compare the nutritional quality of various categories of foods sold on the Italian market, highlighting several critical issues in the ability of some methods to deliver to the consumers the information useful for improving food choices and habits.

Touvier et al. proposed to develop adapted labels able to cover different dimensions potentially affecting the health impact of foods, including the nutritional one (i.e., Nutri-Score), the ultra-processed one (e.g., black band surrounding the Nutri-Score) and the organic dimension by the “organic” logo.

Again, van der Bend et al. described the development and validation process of the Nutri-Score algorithm, suggesting more research on its validity and applicability within the European context.

The work by Saavedra-Garcia et al. investigated changes in marketing strategies (i.e., marketing techniques, health claims, and nutritional claims) on the packaging of foods typically consumed by children before and after the mandatory front-of-package warning label implementation in Peru. Authors found an increase in marketing techniques in “high-in” products, probably used by the industry to reduce the impact of the new FOP on food choices and sales.

Caballero et al. investigated whether eating contexts influence how the mandatory nutrient warning labels in Chile, affect the decision process and selection during food choice. Researches show a rise in the efficacy of this label to improve healthy food choices in a healthy eating context (i.e., when participants were instructed to select the food that they would eat to stay healthy), but not in other eating contexts.

Liao and Yang studied the knowledge, attitude and practice on nutrition labeling in the Chinese population. Authors found a positive attitude toward nutrition labeling in most people, although the awareness and utilization rates were very low. This suggests that the knowledge of nutrition labeling does not directly support the practice.

Finally, Lee et al. examined the diet quality of Canadian adults using a dietary index system aligned with the Canadian FOP regulations compared with other front-of-pack labeling systems and dietary guidelines. Authors observed that the Canadian FOP rates the dietary quality of Canadian adults to be healthier than other systems, however a moderate-to-low agreement with other systems (e.g., Nutri-score, Dietary Approaches to Stop Hypertension and Canada's Food Guide) was found.

The Topic Editors thank all the authors contributing to the Research Topic, with the hope that it will foster ascientific discussion on the FOP useful to better understand how FOP labeling can help consumers to make conscious food choices and the food industry to provide scientifically correct and nutritionally balanced choices to the consumer.

## Author contributions

DM wrote the first draft of the manuscript. MS and JS-S finalize the manuscript. All authors contributed to the article and approved the submitted version.

